# Wavelet-Guided Multi-Scale ConvNeXt for Unsupervised Medical Image Registration

**DOI:** 10.3390/bioengineering12040406

**Published:** 2025-04-11

**Authors:** Xuejun Zhang, Aobo Xu, Ganxin Ouyang, Zhengrong Xu, Shaofei Shen, Wenkang Chen, Mingxian Liang, Guiqi Zhang, Jiashun Wei, Xiangrong Zhou, Dongbo Wu

**Affiliations:** 1 School of Computer, Electronics and Information, Guangxi University, Nanning 530004, China; xjzhang@gxu.edu.cn (X.Z.);; 2 Department of Electrical, Electronic and Computer Engineering, Gifu University, Gifu 501-1193, Japanzhou.xiangrong.n6@f.gifu-u.ac.jp (X.Z.); 3 Department of General Surgery, The Fourth Affiliated Hospital of Guangxi Medical University, Liuzhou 545005, China; 4 Department of Gastrointestinal, Metabolic and Bariatric Surgery, Ruikang Hospital Affiliated to Guangxi University of Chinese Medicine, Nanning 530011, China

**Keywords:** non-rigid medical image registration, unsupervised deep learning, Haar wavelet, ConvNeXt

## Abstract

Medical image registration is essential in clinical practices such as surgical navigation and image-guided diagnosis. The Transformer architecture of TransMorph demonstrates better accuracy in non-rigid registration tasks. However, its weaker spatial locality priors necessitate large-scale training datasets and a heavy number of parameters, which conflict with the limited annotated data and real-time demands of clinical workflows. Moreover, traditional downsampling and upsampling always degrade high-frequency anatomical features such as tissue boundaries or small lesions. We proposed WaveMorph, a wavelet-guided multi-scale ConvNeXt method for unsupervised medical image registration. A novel multi-scale wavelet feature fusion downsampling module is proposed by integrating the ConvNeXt architecture with Haar wavelet lossless decomposition to extract and fuse features from eight frequency sub-images using multi-scale convolution kernels. Additionally, a lightweight dynamic upsampling module is introduced in the decoder to reconstruct fine-grained anatomical structures. WaveMorph integrates the inductive bias of CNNs with the advantages of Transformers, effectively mitigating topological distortions caused by spatial information loss while supporting real-time inference. In both atlas-to-patient (IXI) and inter-patient (OASIS) registration tasks, WaveMorph demonstrates state-of-the-art performance, achieving Dice scores of 0.779 ± 0.015 and 0.824 ± 0.021, respectively, and real-time inference (0.072 s/image), validating the effectiveness of our model in medical image registration.

## 1. Introduction

Medical image registration (MIR) is pivotal in current clinical practice, such as surgical navigation and Computer-assisted diagnosis [1]. MIR can be categorized into rigid registration for rigid tissues and non-rigid registration (or deformable registration) for soft tissues requiring nonlinear spatial adaptation [2]. Compared to simpler rigid registration, traditional non-rigid registration typically relies on instance-specific optimization of predefined deformation models and objective functions to achieve nonlinear spatial mapping, which limits their generalizability across diverse clinical scenarios [3]. Furthermore, the high computational overhead and complex parameter optimization inherent to traditional methods renders them impractical for latency-sensitive clinical environments, such as surgical navigation requiring rapid responses.

In the last decade, deep learning-based registration methods have surpassed traditional MIR in complex deformation modeling, efficiency, and robustness by leveraging large-scale medical image data and powerful feature learning capabilities, replacing instance-specific optimization with a unified global objective function during training to learn universal deformation field mappings [4,5,6,7]. Recent research has shifted focus toward unsupervised registration frameworks that eliminate dependence on ground-truth deformation fields(typically generated by traditional registration methods) in supervised neural network training methods [8]. These unsupervised approaches substantially alleviate the burden of manual data annotation while demonstrating enhanced computational efficiency and registration accuracy compared to traditional registration methods.

Current deep neural network architectures for MIR predominantly use standard convolutional neural networks (CNNs) and Transformers. The advent of Vision Transformer (ViT) [9] and Swin Transformer [10] has established Transformer as a dominant backbone across computer vision tasks—including MIR by outperforming conventional CNNs through superior global context modeling. A core strength of Transformer lies in its self-attention mechanism, which models images as sequences of patch tokens to effectively capture long-range spatial dependencies. However, the quadratic computational complexity of the global self-attention mechanism relative to input size leads to exorbitant computational overhead and a surge in parameters when applying Transformer architectures to high-resolution 3D medical imaging data (e.g., MRI/CT), posing a fundamental conflict with the resource-constrained requirements of clinical deployment scenarios. Furthermore, the weak inherent inductive biases of Transformer require both extensive training data and model scaling (>30 M) to attain peak performance [11,12]. While such large datasets are common in natural images, medical imaging data (particularly annotated 3D volumes) remain scarce due to privacy constraints and annotation complexity. Recently, ConvNeXt [13] is introduced entirely from convolutional modules, through leveraging the superior architecture of transformer for improvement, retains the inherent inductive biases of convolutions while maintaining the efficiency and the number of parameters of standard CNNs. ConvNeXt achieves parity with Transformer in accuracy and robustness while offering superior scalability, demonstrating potential as a backbone network for MIR.

Deep learning-based MIR methods predominantly adopt U-Net architectures [14] with skip connections for weight sharing. Its encoder-decoder structure combines progressive downsampling and upsampling, capturing global context and local details to achieve voxel-level alignment. However, traditional downsampling methods (e.g., max pooling [15] and strided convolutions [16]) reduce image resolution at the cost of spatial information loss (particularly in small structures and edges). Although upsampling (e.g., trilinear interpolation [17], transposed convolution [18]) restores image dimensions, it lacks the capacity to model high-frequency details, failing to recover information lost during downsampling and affecting registration accuracy. Wavelet transform [19] is a multiscale analysis framework that decomposes signals into frequency-localized components while preserving temporal/spatial localization. The theory and application of wavelet transforms have been extensively studied in image processing tasks like decomposition [20] and compression [21]. Its lossless decomposition property grants inherent advantages for downsampling with spatial information preservation—a property particularly advantageous for medical image processing. Dynamic upsampling adaptively adjusts upsampling strategies according to local anatomical context and task requirements, enabling more precise local detail sampling and better restoration of medical image topology compared to fixed-strategy traditional upsampling.

Based on the above analysis, current methods still exhibit several unresolved issues: Firstly, traditional downsampling discards partial information during the process of reducing spatial resolution, which may result in the difficulty of the network in registering key anatomical structural details. Additionally, fixed interpolation strategies during upsampling are insufficient to accurately recover high-frequency information, thus compromising registration accuracy. Secondly, while Transformer-based methods demonstrate advantages in capturing long-range dependencies, their high computational cost struggles to meet real-time requirements. In contrast, conventional convolutional approaches maintain computational efficiency but exhibit limited registration accuracy. Based on these empirical facts, we propose WaveMorph, a novel medical image registration architecture that combines the benefits of multi-scale lossless wavelet decomposition with ConvNeXt architecture and introduces a lightweight dynamic upsampling module. This design minimizes the distortion of key anatomical structures during downsampling and upsampling in the encoding-decoding stages, enhancing registration precision and real-time performance. WaveMorph achieves state-of-the-art performance against baselines comprising Transformer-based models, convolutional networks, and traditional methods. The contributions of this work are fourfold:

1. WaveMorph: We propose a frequency-spatial co-optimization framework for unsupervised non-rigid medical image registration by integrating wavelet transforms and ConvNeXt;
2. MSWF: We design a novel Multi-Scale Wavelet Feature Fusion downsampling module that leverages Haar wavelet decomposition to preserve spatial information across 8 frequency sub-bands, fused via ConvNeXt-optimized multiscale kernels;
3. Dysmaple: We innovatively introduce the lightweight dynamic upsampling module, originally used in image super-resolution, into the medical image registration field. It addresses the issue where traditional upsampling methods often lead to blurring or distortion of key anatomical structures during registration. Extensive experiments in Section 4.4 show that the dynamic upsampling module effectively improves registration accuracy and robustness;
4. State-of-the-art results: We extensively validated our model on inter-patient registration and atlas-to-patient brain MRI registration tasks. WaveMorph achieves superior Dice scores (0.779 ± 0.015 for atlas-to-patient MIR; 0.824 ± 0.021 for inter-patient MIR) and real-time inference (0.072 s/image), outperforming all competing methods in accuracy and efficiency.

## 2. Related Work

### 2.1. Medical Image Registration

Traditional non-rigid medical image registration methods, such as SyN [22], NiftyReg [23], deedsBCV [24], and LDDMM [25], iteratively optimize objective functions at the instance level (given moving-fixed image pairs) to estimate voxel-wise dense nonlinear deformation fields for anatomical correspondence. The objective function of traditional non-rigid MIR is formalized as:

(1) $$\begin{array}{cc} \hat{\emptyset } & =\underset{\emptyset }{\mathrm{argmin}}\;L\left(f,m\circ \emptyset \right) \end{array}$$

(2) $$\begin{array}{cc} \; & =\underset{\emptyset }{\mathrm{argmin}}\;L_{\mathrm{sim}}\left(f,m\circ \emptyset \right)+\lambda L_{\mathrm{reg}}\left(\varphi \right), \end{array}$$

where f:Ω→R and m:Ω→R denote the fixed and moving images, respectively, while $\emptyset :R^{d}\to R^{d}$ denotes the deformation field mapping from *m* to *f*, with registration achieved by minimizing the difference between *f* and m∘∅. $L_{reg}\left(•\right)$ is the smoothness term, with λ acting as the balancing parameter, preventing the network from excessively optimizing image similarity at the cost of introducing unnecessary folds.

Deep learning-based deformable image registration methods optimize the energy function from training datasets to learn the global representation of image registration. For unseen medical images, the network can directly output the deformation field function for a given image pair. These methods can be classified into supervised models and unsupervised models.

Supervised models [26,27,28,29] often obtain the true ground truth labels corresponding to the images to be registered through classical methods and train the model to reproduce the deformation field. The performance of such models often heavily depends on the accuracy of the ground truth deformation fields, which are usually expensive to obtain.

Unsupervised (or “self-supervised”) models [4,5,30,31,32,33] do not need ground-truth deformation fields during the training process. The models are trained by optimizing the contrast differences between image pairs and applying the spatial transformation function [34] to warp the moving image. In some cases, unsupervised learning outperforms supervised learning. For example, single-stream end-to-end registration methods represented by VoxelMorph [4], concatenates the moving and fixed images as two n-dimensional images into a single input, and a convolutional neural network outputs the spatial mapping from the moving image to the fixed image. Additionally, some studies have proposed multi-resolution frameworks for medical image registration [35,36] and GAN-based frameworks [5].

Methods employing standard convolution as the network backbone demonstrate significant advantages in computational efficiency and local feature extraction. However, the limited receptive field of conventional convolution kernels 3×3×3 constrains their ability to model spatial relationships between remote voxels in registration image pairs, thereby restricting deformable registration accuracy. Methods represented by TransMorph [33] address the limitations of standard convolutions in establishing long-range spatial correspondences by incorporating transformers into medical image registration, leveraging the advantages of self-attention mechanisms. However, their high computational costs hinder clinical deployment feasibility and fail to meet the essential requirements of low computational burden and rapid inference in medical applications. This limitation is particularly evident in resource-constrained embedded devices commonly used in surgical navigation systems. Meanwhile, the complex architecture of Transformers makes their decision-making process in attention weight allocation challenging to trace, while high interpretability remains crucial for clinical adoption. The ConvNeXt architecture incorporates design concepts from Vision Transformers (ViT), utilizing depth-wise and point-wise convolutions for construction. Compared to standard convolution operations, it demonstrates enhanced capabilities in feature representation, long-range modeling, and computational efficiency.

### 2.2. Wavelet Transform in Deep Learning

Discrete Wavelet Transform (DWT) serves as a cornerstone signal processing technique, extensively utilized for multiscale decomposition and reconstruction in medical image analysis. Recently, have introduced DWT into neural network architectures to enhance feature representation capabilities in diverse vision tasks such as image classification, super-resolution, and denoising. Xu et al. [37] introduced a simple wavelet downsampling method using Haar wavelet transforms to reduce feature map spatial resolution while preserving as much information as possible. Fujieda et al. [38] introduced supplementing the loss components of multi-resolution analysis using wavelet transforms, incorporating them as additional modules in the overall architecture. As described by Luo et al. [39], a novel wavelet synthesis network architecture enables the rapid generation of high-resolution disparity maps. However, the application of wavelet theory in deep learning-based image registration remains limited. This is primarily because wavelet transformation decomposes images into multiple frequency sub-band components, which may introduce additional complexity to the registration process. For instance, 3D medical images subjected to wavelet decomposition yield eight sub-bands containing distinct spatial and frequency domain information. The optimal strategy for effectively integrating low-frequency (global structural) and high-frequency (boundary or detail) sub-band information to guide feature extraction and spatial transformation during registration remains an open research challenge. Meanwhile, current models based on CNNs or Transformers primarily operate in the spatial domain when processing images. How to better integrate the frequency-domain characteristics from wavelet transforms with spatial features remains an area requiring further investigation.

Current mainstream deep learning-based medical image registration methods (e.g., VoxelMorph [4], TransMorph [33]) predominantly focus on innovations in network backbones or model architectures, typically employing standard downsampling operations (e.g., max pooling and strided convolution). Although offering lower computational complexity, these methods overlook the irreversible loss of high-frequency information (e.g., tissue boundaries, fine anatomical structures) caused by local neighborhood feature aggregation during downsampling, which ultimately compromises model performance. Foremost among these concerns is that input distortions can precipitate topological discontinuities (e.g., folding artifacts) within deformation fields, resulting in physically implausible morphological transformations.

## 3. Methods

### 3.1. Datasets and Preprocessing

To validate the effectiveness of the proposed method, we employed two datasets to evaluate two commonly used tasks in medical image registration. It includes atlas-to-patient registration tasks and inter-patient registration tasks, involving over 1000 T1-weighted brain MRI scans. First, for the atlas-to-patient brain MRI registration task, using the public dataset(IXI) evaluates the performance of the proposed model. The IXI dataset comprises 600 MRI brain scans obtained from normal healthy subjects. Based on the IXI dataset, 576 T1-weighted brain MRI scans from healthy subjects were selected as fixed images, with atlas brain MRI obtained from CycleMorph serving as moving images. The dataset was partitioned into training (403 cases), validation (58 cases), and test sets (115 cases) following a 7:1:2 ratio.

For the more clinically challenging inter-patient registration task, 414 T1-weighted images from the OASIS [40] dataset were utilized. The dataset was partitioned into 394 training cases and 20 test cases according to the TransMorph benchmark experimental protocol (because of the unavailability of an official independent test set). By randomly selecting training set samples as fixed images and pairing them with moving images, while applying image role reversal (i.e., swapping moving/fixed images) to generate dual training samples (i.e., 394 registration pairs) for enhanced model generalization.

All MRI data underwent standardized preprocessing: isotropic resampling 1×1× 1 mm^3^ using Freesurfer [41], AC-PC aligned affine spatial normalization, BET (Brain Extraction Tool) skull stripping, and uniform cropping to 160 (sagittal) × 192 (coronal) × 224 (axial) voxel dimensions. The registration performance evaluation employs a gold standard based on the automated anatomical segmentation of Freesurfer. For the IXI test set, 30 fine-grained anatomical structure labels covering white matter parcellations are used, while the OASIS test set is extended to include 35 anatomical structures incorporating deep nuclei such as the hippocampus and amygdala. These segmentation maps are used solely for assessing registration performance. The experimental design rigorously adheres to the standardized preprocessing pipeline of TransMorph, ensuring fairness in cross-method comparisons.

### 3.2. Implementation Details

We compared WaveMorph with various registration methods that previously demonstrated state-of-the-art registration performance, including three traditional iterative optimization-based methods and three deep learning-based methods. The hyperparameters of all methods were set based on related work and empirical experience to balance registration accuracy and runtime.

WaveMorph was implemented using the PyTorch 2.3.1 framework and CUDA 11.8 on a PC equipped with an NVIDIA RTX 4090 GPU. All models uniformly employed the Adam optimizer with a fixed training schedule of 500 epochs, a learning rate of $1\times 10^{-4}$, and a batch size of 1. During training, experimental setups were differentially configured based on the distinct characteristics of various registration tasks. For the atlas-to-patient brain MRI registration task, data augmentation employing random tri-axis flipping (probability *p* = 0.5) was adopted to enhance the generalization ability for anatomical symmetry. The similarity metric employed normalized cross-correlation loss (NCC: (12)), calculating intensity covariance through a local window (9 × 9 × 9 voxels) to enhance robustness. For inter-patient registration tasks, spatial transformation augmentation was disabled to preserve the original anatomical topology, while mean squared error loss (MSE: (11)) was employed to maximize image alignment accuracy. Both tasks incorporated a diffusion regularization term, with the regularization hyperparameter λ set to 1 (atlas-to-patient registration) and 0.02 (inter-patient registration), respectively. The former reinforces deformation field smoothness to mitigate atlas noise interference, while the latter relaxes constraints to preserve individual anatomical variations. To better demonstrate the architectural advantages of WaveMorph, the experiments strictly maintained consistency in experimental parameters and loss functions with TransMorph.

### 3.3. WaveMorph Architecture for Non-Rigid Registration Network

ConvNeXt retains the inductive bias inherent to convolutional neural networks, endowing it with superior generalization capability compared to Transformer architectures when trained on small-sample medical datasets. Simultaneously, by modeling visual characteristics such as non-sequential processing, rotation, and translation invariance, this approach significantly enhances spatial relationship analysis capabilities for anatomical structures. Although ConvNeXt exhibits weaker long-range modeling capability compared to the global attention mechanisms of Transformer, its strategic adoption of enlarged convolutional kernels (7×7×7 ) effectively expands receptive fields, thereby indirectly strengthening cross-region feature dependency modeling. Unlike conventional CNNs that rely on batch statistical dependencies through BatchNorm, the LayerNorm of ConvNext employs channel-wise standardization, eliminating sensitivity to batch dimensions, thereby demonstrating enhanced robustness and generalization capability in small-batch medical image registration tasks.

**Figure 1:**
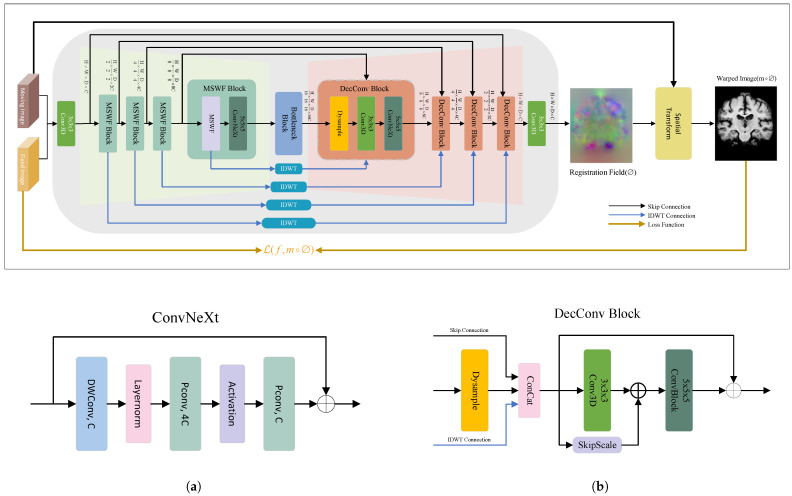
The overall framework of the proposed WaveMorph image registration model. Given an input image pair of the moving image *m* and the fixed image *f*, the WaveMorph network outputs the deformation field, and the spatial transformation function warps *m* into the warped image m∘∅, minimizing the discrepancy between *f* and m∘∅. (**a**) The architecture of the ConvNeXt module. DWConv denotes Depthwise Convolution, PConv denotes Pointwise Convolution, Activation denotes the Non-linear Activation Layer, and C denotes the Channel Dimension. (**b**) The architecture of the decoder convolution(DecConv) block. Dysample denotes the Lightweight Dynamic Upsampling Layer, skipscale denotes skip connections with learnable parameters.

Figure 1 illustrates the proposed single-stream unsupervised image registration network, WaveMorph. The moving image *m* and fixed image *f* have been pre-aligned affine in the data preprocessing stage, enabling the network to focus on nonlinear spatial mapping between voxels. WaveMorph employs a U-Net-inspired encoder-decoder architecture. The single-channel 3D medical images *m* and *f* are concatenated channel-wise into a tensor $I:\Omega \to R^{2C\times H\times W\times D}$, where *C*, *H*, *W* and *D* denote channels, height, width, and depth, respectively. A standard convolutional operation is applied to mix and share features, forming implicit strength distribution differences (nonlinear mapping relationships).

The encoder comprises four cascaded Multi-Scale Wavelet Feature Fusion (MSWF) Blocks, each combining: (1) an MSWF module for lossless downsampling and (2) a ConvNeXt-based feature extraction module (ConvNeXt). Progressive downsampling in the encoder yields a bottleneck feature map $F\in R^{\left(8\times 2^{4}\right)\times \frac{H}{16}\times \frac{W}{16}\times \frac{D}{16}}$. Subsequently, WaveMorph uses a bottleneck block to capture long-range spatial correlations while preserving the feature map’s resolution $\frac{H}{16}\times \frac{W}{16}\times \frac{D}{16}$. The decoder incorporates four consecutive Decoder Convolution Blocks (DecConv Blocks), consisting of continuous dynamic upsampling layers, standard convolution, and ConvNeXt with different kernel sizes, along with residual connections, for feature decoding and image resolution recovery. During the decoder phase, WaveMorph follows the U-Net-style skip connections, where each upsampled feature map is concatenated with the corresponding decoder feature map through skip connections to provide multi-level feature mapping for the decoder.

To overcome single-scale limitations in standard skip connections, we augment them with multi-scale convolution-enhanced inverse discrete wavelet transform (IDWT) features, preserving cross-frequency anatomical details. Finally, a standard convolution layer generates a deformation field $\emptyset \in R^{3\times H\times W\times D}$ at the original resolution, representing voxel-wise displacements in three orthogonal axes. The spatial transformer warps *m* into m∘∅, and the similarity between *f* and m∘∅ is evaluated using (2) to achieve precise anatomical alignment.

### 3.4. Multi-Scale Wavelet Feature Fusion Module

The three-dimensional discrete wavelet transform (DWT) orthogonally decomposes an input image into eight subbands at half the original resolution using eight filters composed of low-pass *L* and high-pass *H* filters (i.e., $f_{lll}$, $f_{llh}$, $f_{lhl}$, $f_{lhh}$, $f_{hll}$, $f_{hlh}$, $f_{hhl}$, $f_{hhh}$) along height *H*, width *W* and depth *D*, each containing distinct spatial-frequency information. Thus, the input image is decomposed into a pure low-frequency component LLL, a pure high-frequency component HHH, and six components containing both high- and low-frequency mixed bands LLH, LHL, LHH, HLL, HLH, HHL. In this study, we use the efficient and simple Haar wavelet as the basis function. However, other basis functions (e.g., Daubechies) are also applicable, though they may increase the computational cost. Due to the orthogonal property of DWT, the image can be accurately reconstructed via the inverse discrete wavelet transform (IDWT), even after convolutional linear transformations.

**Figure 2:**
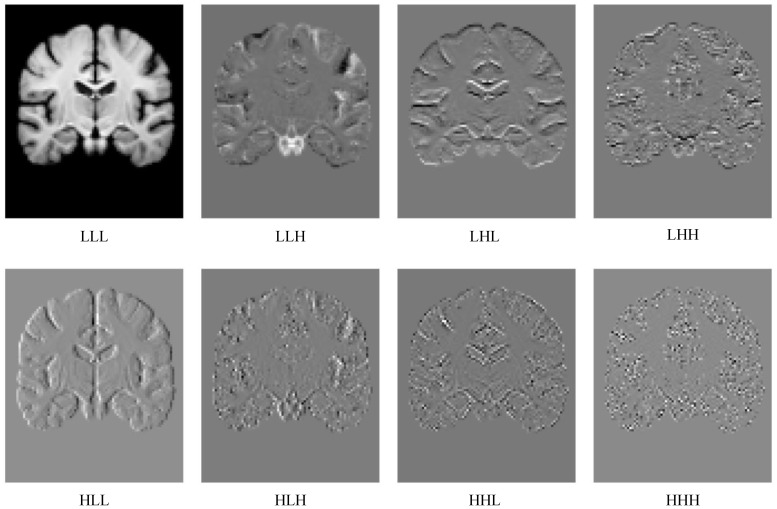
The eight subband diagrams decomposed by Haar wavelet transform, where L denotes the low-pass filter and H denotes the high-pass filter.

Chen et al. [42] proposed convolving inputs by initially separating high- and low-frequency components and enabling information exchange between them. Although the method is not related to wavelet transforms, it demonstrated the benefits of separately performing convolutions on the low-frequency component and the high-frequency component to obtain more informative feature maps. Inspired by this, we creatively integrate the properties of Discrete Wavelet Transform (DWT) to propose the Multi-Scale Wavelet Feature Fusion downsampling module, as shown in Figure 2. For the eight subband images obtained after the wavelet transform, we implement differentiated feature enhancement strategies based on the different informational characteristics carried by each subband feature.

**Figure 3:**
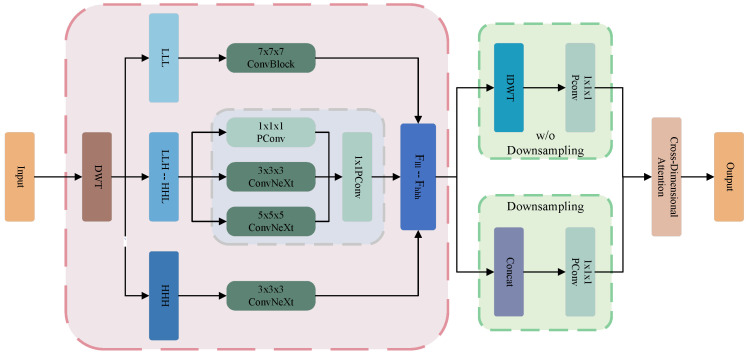
The architecture of the proposed Multi-Scale Wavelet Feature Fusion Module(MSWF), where DWT and IDWT denote Discrete Wavelet Transform and Inverse Discrete Wavelet Transform, LLL−HHH denote the eight subbands, $F_{lll-hhh}$ denote the enhanced features, and the ConvNeXt architecture is detailed in Figure 1a. The proposed module implements a differentiated feature enhancement strategy for the eight Haar wavelet subbands. Convolutional kernels with adaptive receptive fields process each subband in parallel, followed by feature fusion. Two distinct strategies (green blocks) are defined based on the inclusion/exclusion of downsampled feature maps, where “w/o Downsampling” denotes the absence of downsampling. A cross-dimensional attention layer subsequently refines the features.

As shown in Figure 3, for the pure low-frequency subband images LLL containing global information, we use a ConvNeXt block with large 7×7×7 kernels for feature extraction. The large convolution kernel has a larger receptive field, enabling it to capture the overall structure and global information of the image, which helps enhance the modeling ability of global deformations during the registration process. For the pure high-frequency subband images HHH, encoding local anatomical details is processed via 3 × 3 × 3 convolutions to preserve fine-grained details essential for resolving subtle registration misalignments. For the six hybrid subband images LLH−HHL, we use convolution kernels with three different scales (kernel sizes of 1, 3, and 5) for feature extraction. These subband images contain mixed-frequency information of the image in different dimensions, and the multi-scale convolution kernels are capable of capturing features at different scales. Features from the three kernel scales are channel-concatenated and fused via pointwise convolution, yielding enriched representations with global awareness and adaptability to irregular structures. For the eight enhanced subband images, MSWF operates in two modes (Figure 3, green boxes): (1) downsampled feature output (discussed here) and original-resolution output (detailed in Section 3.5).

In downsampling mode, MSWF framework obtains eight enhanced features $F_{lll-hhh}$ through three distinct feature enhancement strategies, compresses the channel dimension to the output dimension using a pointwise convolution. Due to the separate feature enhancement applied to the eight subband images after wavelet transform, the advantages of convolutional feature sharing exist only within the individual subbands, and the lack of cross-subband information sharing hinders the model’s expressive power. To address this, MSWF introduces a four-branch cross-channel interactive attention layer (CDA), which strengthens the fusion of channel and spatial information between different sub-images, ensuring full exchange and adaptive adjustment of frequency information, thereby improving the stability of training and the generalization ability of the model. A complete MSWF can be represented as:

(3) $$\begin{array}{cc} \; & \left[LLL,LLH,LHL,LHH,HLL,HLH,HHL,HHH\right]=DWT\left(F_{in}\right) \end{array}$$

(4) $$\begin{array}{cc} \; & F_{lll}=Conv_{7}\left(LLL\right) \end{array}$$

(5) $$\begin{array}{cc} \; & F_{hhh}=Conv_{3}\left(HHH\right) \end{array}$$

(6) $$\begin{array}{cc} \; & F_{llh-hhl}=PConv\left(Concat(Conv_{k}\left(LLH-HHL\right))\right),k\in \left\{1,3,5\right\} \end{array}$$

(7) $$\begin{array}{cc} \; & F_{out}=CDA\left(PConv(Concat\left(F_{lll},F_{llh},F_{lhl},F_{lhh},F_{hll},F_{llh},F_{hhl},F_{hhh}\right))\right) \end{array}$$

where $F_{in}$ and $F_{out}$ denote the input and output feature maps, DWT denotes the three-dimensional discrete wavelet transform, [LLL−HHH] denotes the eight subband images with halved resolution after wavelet transformation, Conv refers to the ConvNeXt Block, with the subscript indicating the kernel size, $F_{lll-hhh}$ denotes enhanced features from ConvNeXt Blocks, PConv refers to pointwise convolution, and CDA refers to the cross-dimensional attention layer.

### 3.5. Bottleneck Block

The traditional encoder progressively reduces spatial resolution via downsampling, achieving dual benefits: (1) reduced computational and memory overhead and (2) expanded effective receptive fields for fixed-kernel convolutions. At the encoder terminus, we propose an innovative bottleneck block to enhance these benefits, maximizing the receptive fields at minimal computational cost to further model long-range spatial dependencies. Bottleneck Block combines two sequential MSWF Blocks that maintain original resolution. Specifically, as shown in Figure 3 (green block, w/o downsampling branch), input features are decomposed into eight half-resolution subbands images via DWT. These subband images are processed in parallel through multi-scale ConvBlocks to enhance the features, followed by an inverse wavelet transform to restore the resolution to match the input feature map. The features are then fused through an attention mechanism and further extracted using large-kernel ConvNeXt Block, ensuring comprehensive utilization of global information in the network, ultimately producing the output feature map.

The entire bottleneck block can be viewed as two miniature U-Net structures. The bottleneck block enables the fine extraction and fusion of image features at multiple scales, especially for features related to global deformations, under maximal theoretical receptive fields while maintaining channel counts and resolution. The full-resolution MSWF module (Figure 3, pink block) follows Equations (3)–(6) to generate eight convolution-enhanced features.

(8) $$\begin{array}{cc} \; & F_{idwt}=IDWT\left(F_{lll},F_{llh},F_{lhl},F_{lhh},F_{hll},F_{llh},F_{hhl},F_{hhh}\right) \end{array}$$

(9) $$\begin{array}{cc} \; & F_{out}=Conv_{7}\left(CDA(PConv\left(F_{idwt}\right))\right) \end{array}$$

where IDWT denotes the three-dimensional inverse discrete wavelet transform, while $Conv_{7}$ refers to the ConvNeXt Block with a kernel size of 7×7×7. MSWF will restore the eight convolution-enhanced features to the original resolution using Equations (8) and (9), followed by further feature refinement.

### 3.6. Lightweight Dynamic Upsampling Module

In deep learning-based MIR, upsampling modules are typically located in the decoder to restore spatial resolution and enable hierarchical feature aggregation across scales. Its accuracy and efficiency directly impact the accuracy of the registration result and computational resource consumption. Traditional upsampling methods (e.g., nearest-neighbor interpolation and trilinear interpolation) rely on fixed interpolation rules, which struggle to accommodate the complex anatomical structures in medical images (e.g., subtle anatomical features or tumor boundaries). This often causes edge blurring or jagged artifacts after registration, reducing the accuracy of registration.

To alleviate such issues and adapt to computationally constrained medical scenarios, we introduce the lightweight dynamic upsampling method DySample [43] from the image super-resolution field into the MIR task. DySample bypasses traditional dynamic convolution by modeling geometric information via dynamic sampling points without requiring high-resolution guided features or complex sub-networks. This only introduces a minimal number of parameters (<1 k additional parameters) to perform dynamic sampling. Specifically, DySample generates sampling offsets via linear projection and resamples using PyTorch’s grid_sample function. Key implementations: (1) Bilinear initialization ensures zero-offset consistency; (2) Dynamic Scope Factor limits offset ranges to prevent overlap artifacts; (3) Grouped upsampling enables channel-wise adaptive offsets. Extensive experiments in Section 4.4 show that the introduction of DySample significantly improves the registration accuracy of medical images, with negligible impact on GPU memory usage and training time.

### 3.7. Spatial Transformation Function

The optimization objective of WaveMorph is to minimize the dissimilarity between the warped image m∘∅ and *f*. The spatial transformer network (STN) [34] provides differentiable geometric transformations to compute m∘∅ to generate the warped image. For medical images, this can be defined as a linear interpolation of the eight neighboring voxel values around each voxel:

(10) $$m\circ \phi \left(p\right)=\sum_{q\in Z(p^{\prime })}m\left(q\right)\prod_{d\in \{x,y,z\}}\left(1-\right|p_{d}^{\prime }-q_{d}\left|\right)$$

Let $p^{\prime }=p+u\left(p\right)$ define the deformed position, where $u\left(p\right)\in R^{3}$ is the displacement vector estimated by the network, and $Z(p^{\prime })$ denotes the eight neighboring voxels of $p^{\prime }$.

### 3.8. Loss Functions

The loss function for network training remains consistent with conventional approaches, comprising two components: an image similarity metric between the input volume and registration domain and a regularization term enforcing spatial smoothness of the deformation field.

Our experimental framework utilizes two established similarity metrics for unsupervised model evaluation. The primary metric, Mean Squared Error (MSE), calculates the mean of squared voxel differences (applicable to moving/fixed image pairs with aligned contrast and intensity profiles), where $p_{i}$ denotes voxel positions and Ω defines the spatial domain:

(11) $$\;MSE=\frac{1}{\left|\Omega \right|}\sum_{i\in \Omega }{\left(I_{fixed}\left(p_{i}\right)-I_{moved}\left(p_{i}\right)\right)}^{2}$$

The secondary metric, Local Normalized Cross-Correlation (LNCC), quantifies similarity through localized window comparisons across image volumes, offering enhanced robustness against intensity and contrast discrepancies:

(12) $$\begin{array}{cc} \; & NCC\left(f,\left[m\circ \varphi \right]\right)=\frac{\sum_{p_{i}\in \Omega }\left(f\left(p_{i}\right)-\mu_{f}\right)\left(\left[m\circ \varphi \right]\left(p_{i}\right)-\mu_{m\circ \varphi }\right)}{\sqrt{\sum_{p_{i}\in \Omega }{\left(f\left(p_{i}\right)-\mu_{f}\right)}^{2}\sum_{p_{i}\in \Omega }{\left(\left[m\circ \varphi \right]\left(p_{i}\right)-\mu_{m\circ \varphi }\right)}^{2}}} \end{array}$$

where $\mu_{f}$ and $\mu_{m\circ \varphi }$ denote the average voxel intensity in a localized $n^{3}$ window around voxel *p*. The parameter n=9 was employed throughout our experiments.

## 4. Results

### 4.1. Evaluation Metrics

This study employs a multi-dimensional quantitative metric system to evaluate registration performance. Specifically, leveraging anatomical segmentation consistency, we compute the Dice Similarity Coefficient (DSC) for 30 (IXI dataset)/35 (OASIS dataset) brain structures, defined as:

(13) $$\mathrm{DSC}=\frac{2|X\cap Y|}{\left|X|+|Y\right|}$$

where *X* and *Y* denote the binarized segmentation masks of the registered moving image and fixed image, respectively. For each registered image pair, per-structure DSC values are calculated, followed by hierarchical statistical methods to derive group-level means and standard deviations.

To evaluate deformation field biomechanical validity, a Jacobian determinant topology metric is applied—spatial differentiation of the deformation field produces Jacobian determinant distributions. We define the Folding Ratio (FR), the percentage of non-positive Jacobian voxels, to measure topological preservation:

(14) $$\mathrm{FR}=\frac{1}{N}\sum_{v\in V}I(|J_{\phi }\left(v\right)\left|\leq 0\right)\times 100\%$$

where *V* denotes the set of all 3D brain voxels, *N* is the total voxel count, and I(…) epresents the indicator function. This metric quantifies local irreversible folding artifacts in the deformation field, with lower values indicating superior topological preservation.

### 4.2. Registration Results

**Figure 4:**
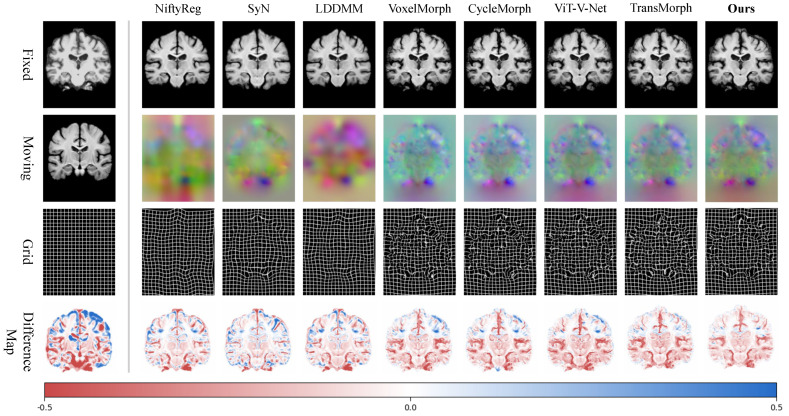
Qualitative comparison of various registration methods on the atlas-to-patient brain MRI registration task. The first column includes the fixed image, moving image, and standard grid. Excluding the first column, the first row displays the warped moving images, the second row visualizes the deformation fields (spatial dimensions x, y, z mapped to RGB color channels, respectively), the third row presents the deformed grids, and the last row shows the absolute difference maps between the warped images and the fixed image.

We conduct a systematic evaluation of WaveMorph across two pivotal brain MRI registration paradigms: atlas-to-patient registration and inter-patient registration. Top-performing models from the validation phase (based on Dice scores) were retained for test-set benchmarking. Figure 4 displays qualitative results from atlas-to-patient brain MRI registration. The first row displays warped images from various registration methods, the second and third rows visualize the deformation fields(the displacement components across three dimensions are encoded into RGB color channels) and the images demonstrating deformation fields applied to a standard grid. Despite lacking explicit diffeomorphic constraints, WaveMorph generates topology-preserving displacement fields with smoother spatial gradients (Figure 4 row 3 and 4) and the lowest absolute registration errors (bottom row). Figure 4 the bottom row visualizes absolute differences between pre-registration warped images and fixed images across baseline methods, with differences normalized to [−0.5, 0.5] and mapped to the RGB gamut. A lighter color intensity indicates lower registration error values. Visual inspection confirms that our method achieves minimal absolute error metrics across all comparative cases, outperforming all baselines in visual fidelity.

**Figure 5:**
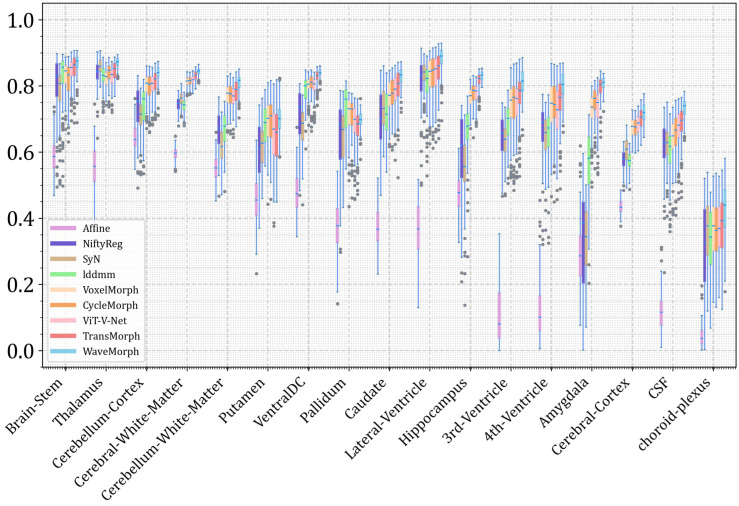
Quantitative comparison of the various registration methods on the atlas-to-patient brain MRI registration task. Boxplots showing Dice scores for different brain MRI substructures using the proposed WaveMorph and existing image registration methods.

Quantitative results (Table 1) demonstrate that WaveMorph achieves state-of-the-art Dice scores and fewer folded voxels compared to non-diffeomorphic deep learning methods in both tasks. In atlas-to-patient registration, WaveMorph achieves a mean Dice of 0.779 ± 0.015, a statistically significant 2.7% improvement (*p* < 0.0001) over TransMorph, the previous state-of-the-art. For inter-patient registration, WaveMorph achieves a mean Dice of 0.824 ± 0.021 (1.5% higher than the best method) while maintaining real-time inference speeds (0.072 s/image). Table 2, WaveMorph significantly outperforms existing methods in terms of inference speed (0.072 s/image), achieving a 2.73 times improvement over the second-place ViT-V-Net (0.197 s/sample). As shown in Figure 5, the Boxplot showing Dice scores for different brain MRI substructures using the proposed WaveMorph and existing MIR methods. Our method achieves higher Dice scores on most anatomical structures, such as lateral-ventricle, third-ventricle and choroid-plexus. Our method demonstrates systematic superiority in registration accuracy for critical anatomical structures: the elevated Dice scores achieved on millimeter-scale anatomical structures (third/fourth ventricles, choroid plexuses) validate the enhanced registration accuracy enabled by MSWF and Dysample modules in preserving and recovering fine-grained features; large-volume anatomical structures including Cerebral Cortex, Cerebellum Cortex, and Thalamus also achieved consistently high Dice scores. Experimental results validate the capability of ConvNeXt superior in large deformation modeling. Notably, WaveMorph demonstrates significantly reduced interquartile range (IQR) across anatomical metrics compared to benchmark methods, indicating enhanced robustness and improved generalization capability. WaveMorph demonstrates superior clinical applicability (4.6 times faster than Transformer-based models) compact parameterization (0.7 M), enhanced robustness, and submillimeter registration accuracy.

### 4.3. Computational and Model Complexity

**Figure 6:**
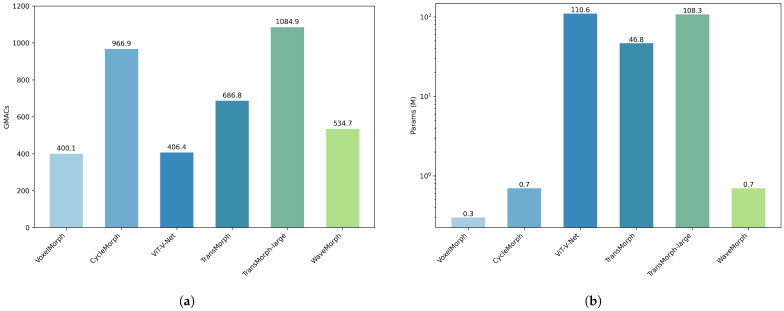
(**a**) Model computational complexity comparisons represented in Giga multiply–accumulate operations (GMACs). (**b**) The number of parameters in each deep-learning-based model. The values are in units of millions of parameters.

As shown in Figure 6, we compare the computational and model complexity of deep learning-based registration models. The experiment uses image sizes consistent with our brain MRI dataset. Transformer-based architectures exhibit prohibitive quadratic computational complexity ($O(n^{2})$), resulting in excessive resource demands (e.g., >30 M parameters). Standard convolution-based registration models have computational complexity comparable to that of Transformer, with trainable parameters typically less than 1 M, but their registration performance is significantly worse. In contrast, WaveMorph exhibits intermediate computational complexity (534.71 GMACs), 40% lower than CycleMorph, while synergizing the efficiency of CNNs with Transformer-like representational capacity. In terms of model complexity (trainable parameters) comparable to CNNs, WaveMorph achieves state-of-the-art accuracy across all tasks with orders-of-magnitude fewer parameters (0.7 M vs. 46.8 M in TransMorph). The architectural advantage of WaveMorphh gives it smaller computational and model complexities than Transformer models, along with superior performance, fully meeting the need for low parameters and low computational load in practical medical environments.

### 4.4. Ablation Studies

We conducted extensive ablation studies to validate the efficacy of each proposed module. MSWF downsampling and DySample upsampling were independently evaluated on atlas-to-patient and inter-patient brain MRI datasets.

As shown in Table 3, we selected three downsampling strategies: max pooling (common in most tasks), PatchMerging reported in SwinTransformer, and the downsampling strategy using Haar wavelet (wavesample) reported by Xu et al. [37]. Since the wavesample was originally applied to 2D natural image segmentation, we extended it to 3D for experimental purposes. Likewise, we selected two upsampling strategies: Nearest neighbor interpolation and Trilinear interpolation. Combining MSWF and DySample yielded 12 module combinations (4 downsamplers × 3 upsamplers) to assess their impact on registration performance. For the network architecture, we adopted the WaveMorph framework, where ConvneXtBlock was used as the feature extraction layer in the encoder, and DecConv Block was used in the decoder. For the bottleneck block without the MSWF module, we used two consecutive ConvneXtBlocks instead.

The experimental results show that when the upsampling module is fixed as Dysample, MSWF outperforms PatchMerging, improving the Dice coefficient by 2.7% in the atlas-to-patient registration task and 0.5% in the patient-to-patient registration task. With MSWF as the fixed downsampling module, Dysample outperforms Nearest neighbor interpolation, improving the Dice coefficient by 2.1% in the atlas-to-patient registration task and 0.9% in the patient-to-patient registration task. The MSWF+DySample configuration achieves state-of-the-art accuracy: 0.779 ± 0.015 (atlas-to-patient) and 0.824 ± 0.021 (inter-patient) mean Dice scores. We observed that by using only the Dysample upsampling module in the WaveMorph framework, we still achieved performance close to or surpassing that of TransMorph. Additionally, when only the MSWF downsampling module and the non-downsampling MSWF module as the bottleneck block were used, better performance than TransMorph was achieved in both registration tasks, further validating the important contribution of the proposed MSWF module to stability and performance.

## 5. Discussion

### 5.1. Analysis of Information Importance

In deep learning architectures, the non-traceable information dissipation caused by conventional pooling operations (e.g., max or average pooling) leads to irreversible feature distortion, particularly compromising the network capacity to preserve the topological integrity of input data. Unlike conventional black-box dimensionality reduction, wavelet transforms provide mathematically invertible downsampling with explicit spectral decomposition, preserving frequency-specific features across subbands to enable clinicians and researchers to trace multiscale feature extraction patterns, thereby enhancing the interpretability of network training dynamics. Specifically, wavelet decomposition separates input images into multi-frequency components: the low-frequency subband preserves global structural information analogous to conventional downsampling, while high-frequency subbands explicitly encode edge or texture features along horizontal, vertical, and diagonal orientations. These high-frequency subbands provide complementary representations for fine-grained anatomical details, including tissue boundaries and small-scale structures, through enhanced edge gradient preservation in multiple orientations. Multi-scale frequency-domain analysis enhances edge, orientation and texture feature perception in medical image registration, reducing CNN registration instability caused by local feature extraction through adaptive spectral decomposition.

CNN-based approaches like VoxelMorph and CycleMorph commonly use max pooling in the downsampling phase, lowering resolution by preserving only local maximum values. Information discarded during this process is irrecoverable and unavailable for network learning, thereby compromising the precision of registration. As shown in Table 3, the MSWF module increases the Dice coefficient by 2.3% compared to MaxPooling. Transformer-based approaches like ViT-V-Net and TransMorph mitigate the information loss associated with MaxPooling by utilizing the PatchMerging strategy. The core idea is to divide the image into multiple patches and then reduce resolution through stitching and compression. While the interference from information mixing caused by this method has minimal impact in natural images, such aliasing significantly affects the topological preservation of anatomical structures in medical image registration tasks, thereby degrading registration performance. As shown in Table 3, the MSWF module increases the Dice coefficient by 2.3% compared to PatchMerging. The success of WaveMorph can be attributed to its unique architectural design. The input image undergoes lossless multi-scale decomposition via Haar wavelet transform, resulting in eight frequency sub-images that capture low-frequency global structures and high-frequency local details. Combining the hierarchical feature extraction capabilities of ConvNeXt under different receptive fields, the encoder achieves complementary enhancement of multi-level frequency and spatial domain information. It effectively reduces the information loss during downsampling in traditional U-Net. Low-frequency sub-images use large convolutional kernels (7×7×7) to capture global deformation features. Containing high- and low-frequency sub-images uses convolutional kernels of different sizes to achieve fusion and enhancement of local and global features. High-frequency sub-images use 3×3×3 convolutions to extract edge details. This differentiated strategy enables the extraction of multi-granularity features, enhancing adaptability to global large-displacement deformations and local subtle deformations. Meanwhile, it avoids feature bias caused by single convolutional kernels and model fragility due to single information sources (In other words, the model has poor generalization ability).

In single-stream non-rigid medical image registration methods based on deep learning, the generation of the final deformation field primarily relies on the decoder to gradually restore the spatial resolution of feature maps through upsampling layers and convolutional operations, mapping the low-dimensional features compressed by the encoder to deformation fields with the same dimensions as the input images. The accuracy of nonlinear spatial registration in resultant images largely depends on the capability of upsampling layers to restore intricate anatomical details present in medical imaging data precisely. Current upsampling layers utilize fixed-strategy upsampling methods. Historically, nearest-neighbor interpolation was predominantly employed, assigning the grayscale value from the input pixel nearest to the target mapped coordinate as the sampled point’s pixel value. In recent 3D imaging studies, existing methods employ trilinear interpolation to mitigate blocking artifacts (e.g., jagged edges or mosaic patterns) and poor spatial continuity inherent in nearest-neighbor interpolation. The core principle involves performing linear interpolation sequentially along three axes, combining the values of eight nearest neighbor voxels through weighted calculation to determine the target voxel’s value. However, trilinear interpolation employs a strategy solely based on linear interpolation within local neighborhoods, which cannot recover details lost in the original image due to insufficient resolution and merely smooths existing data compared to nearest-neighbor interpolation. The objective of image super-resolution is to recover additional high-frequency components through the manipulation of low-resolution image data, targeting the improvement of perceptual image quality. Such as, the reconstruction of high-resolution MRI can clearly display the boundaries of tumors and their relationship with surrounding tissues. This is consistent with the requirements for precise medical image registration. The lightweight DySample upsampling module adjusts sampling locations via dynamic offsets to accommodate the needs of various anatomical regions. During network training, jointly optimized alongside the loss function, the framework enhances resolution recovery while maintaining fidelity to fine-grained details and anatomical features characteristic of medical imaging data. Like Table 3, The introduction of Dysample increased the Dice coefficient by 2.1% for nearest-neighbor interpolation and 1.5% for trilinear interpolation, demonstrating that its enhancement of image detail features plays a critical role in improving registration accuracy.

### 5.2. Convergence and Speed

Compared to Transformer-based models with typically exceeding 40 M learnable parameters, ConvNeXt-based networks achieve superior registration accuracy with under 1 M parameters, while their efficient depthwise convolution operations enable faster inference speed, better satisfying clinical deployment requirements for low computational load and low-latency processing.

During model training, compared to other deep learning-based methods, WaveMorph attained near-peak Dice scores within 50 epochs, while TransMorph required nearly 250 epochs. It demonstrated that the lossless transmission of information throughout the network enables it to learn the spatial correspondence between image pairs faster than competing models. This indicates that WaveMorph exhibits a shorter “transient” phase in the biphasic training [44,45] curves of deep learning models compared to baseline methods. The network rapidly identifies the neighborhood of local minima, and enters a “minimization” phase in subsequent training epochs to search for local minima within this region. The rapid convergence property significantly reduces training time while also conserving computational resources and costs. Notably, WaveMorph employs pure convolutional architecture that maintains performance superiority throughout the training cycle over other standard convolution-based models, despite minimal increases in computational load and parameter count. It indicated that the ConvNeXt architecture is more effective than standard convolutions, thereby significantly improving registration performance.

Table 2 compares the inference time of existing conventional methods with both the training time (min/epoch) and inference time (s/image) of deep learning-based baseline approaches. All methods were implemented on the IXI dataset using identical training and test sets, with computations executed on GPU hardware (some traditional methods are CPU-based). The most and second most time-consuming training methods are the CycleMorph (based on GAN) and TransMorph (based on Transformer), requiring approximately 8 days and 3 days of training time, respectively. Although CycleMorph architecture consists of standard convolutions, its cycle-consistent training requires simultaneous training of multiple networks within a single training cycle, resulting in significant time consumption. The prolonged training time of TransMorph stems from its parameter count being approximately 70 times that of convolutional models, and the GPU memory consumption of Adam optimizer is roughly twice the parameter size (the optimizer incurs an extra memory overhead approximately 140 times greater than that required by standard convolutional architectures). The enormous volume of trainable parameters coupled with intensive GPU memory demands markedly hinder training efficiency. In this paper, we proposed WaveMorph which combines the advantage of an extremely low parameter count compared to convolutional models with kernel optimization techniques, achieving the fastest cyclic training speed and an outstanding inference speed of merely 0.072 s. WaveMorph achieves a 2.7 times improvement in inference speed compared to ViT-V-Net, the fastest baseline method, and a 4.6 times improvement compared to TransMorph, the baseline with the highest registration accuracy. In practical surgical navigation workflows, taking neurosurgical procedures as an example, surgeons need to monitor the positional relationships between surgical instruments and critical surrounding tissues (e.g., nerves and blood vessels) in real time during operations, where timeliness serves as the critical determinant of procedural accuracy and safety. Current deep learning models still exhibit a notable latency of 0.2–0.5 s, even with GPU acceleration. WaveMorph maintains registration latency below 0.1 s, ensuring real-time synchronization between navigational images and patient anatomical structures to prevent visual-motion desynchronization and better meet real-time interaction requirements. The proposed network architecture can be readily integrated with multi-scale strategies, cycle-consistent (GAN), and other training adaptation methods while maintaining compatibility with arbitrary registration loss functions.

## 6. Conclusions

In this paper, we present WaveMorph, a pure convolutional model designed for unsupervised deformable image registration. WaveMorph is a novel neural network that integrates wavelet transform and ConvNeXt. Joint frequency-spatial domain optimization significantly enhances feature representation capabilities, while dynamic upsampling techniques effectively address high-frequency detail modeling deficiencies. Compared with the Transformer, our model demonstrates significant advantages and efficiency in computational and model complexity. This makes WaveMorph a strong candidate for addressing computational resource limitations and enabling real-time clinical applications in practical medical settings.

There are some limitations in our work. First, due to constraints in training time and GPU resource availability, we configured hyperparameters through empirical or baseline-suggested values without extensive grid search optimization. Furthermore, the wavelet transform in this study currently relies on a fixed wavelet basis (Haar) and cannot dynamically learn the optimal transformation through gradient descent. Additionally, this study proposes adopting differentiated feature extraction strategies for multi-scale subbands derived from wavelet decomposition, guided by the information carried by their frequency bands. While MWFS demonstrated strong performance in two benchmark tests, it did not fully account for the dynamic variations in the contribution levels of different frequency bands.

In future research, we plan to explore the following directions: (1) Extending the evaluation of WaveMorph to other organs, such as the lungs, heart, and abdomen, to assess its generalizability; (2) Enhancing training data using image generation strategies or replacing conventional loss functions (e.g., mutual information) to further expand its potential for multimodal registration tasks; (3) Designing learnable wavelet basis functions that adapt to data distributions, enabling improved feature representation and synergistic optimization of frequency-domain representation and spatial deformation; (4) Further lightweighting the model and investigating its deployment on low-power clinical devices, such as mobile PACS systems and edge computing devices, for real-world medical applications.

## Figures and Tables

**Table 1:** Qualitative comparisons between our framework and other methods on the Atlas-to-patient (IXI) and Inter-patient (OASIS) brain MRI registration task. A higher Dice Score indicates more accurate registration results. FR denotes the average percentage of folding voxels in the deformation fields. Lower values indicate greater smoothness. The **bolded** numbers denote the highest scores.

	Atlas-to-Patient MRI		Inter-Patient MRI	
**Model**	**DSC**	**FR**	**DSC**	**FR**
Affine	0.406 ± 0.035	-	0.571 ± 0.053	-
SyN	0.645 ± 0.152	<0.0001	0.769 ± 0.028	<0.0001
NiftyReg	0.645 ± 0.167	0.020 ± 0.046	0.762 ± 0.034	0.020 ± 0.046
LDDMM	0.733 ± 0.126	<0.0001	0.733 ± 0.126	<0.0001
VoxelMorph	0.729 ± 0.129	1.590 ± 0.339	0.787 ± 0.026	1.290 ± 0.319
CycleMorph	0.737 ± 0.029	1.719 ± 0.382	0.793 ± 0.025	1.219 ± 0.362
ViT-V-Net	0.732 ± 0.030	1.554 ± 0.270	0.808 ± 0.023	1.224 ± 0.348
TransMorph	0.752 ± 0.029	1.440 ± 0.303	0.809 ± 0.022	0.390 ± 0.328
Ours	**0.779 ± 0.015**	1.310 ± 0.313	**0.824 ± 0.021**	0.204 ± 0.047

**Table 2:** Average training and inference time for methods used in this work. Note that SyN and NiftyReg were applied using CPUs, while LDDMM and the learning-based methods were implemented on GPU. Inference time was averaged based on test dataset runs. The **bolded** numbers denote the highest scores.

Model	Training (min/epoch)	Inference (s/image)
SyN	-	192.140
NiftyReg	-	30.723
LDDMM	-	66.829
VoxelMorph	4.93	0.430
CycleMorph	21.99	0.281
ViT-V-Net	4.83	0.197
TransMorph	7.56	0.329
Ours	**3.88**	**0.072**

**Table 3:** The ablation study of the WaveMorph model evaluates various existing upsampling and downsampling methods, as well as the MSWF downsampling and Dysample upsampling modules, where up denotes upsampling methods and down denotes downsampling methods. The **bolded** numbers denote the highest scores.

Up	Nearest	Trilinear	Dysample	Atlas-to-Patient MRI		Inter-Patient MRI	
Down				DSC	FR	DSC	FR
maxpooling	✓	×	×	0.747 ± 0.030	1.581 ± 0.329	0.809 ± 0.017	0.167 ± 0.056
	×	✓	×	0.748 ± 0.029	1.578 ± 0.348	0.811 ± 0.019	0.174 ± 0.055
	×	×	✓	0.756 ± 0.028	1.591 ± 0.355	0.815 ± 0.018	0.222 ± 0.062
patchmerging	✓	×	×	0.748 ± 0.030	1.511 ± 0.321	0.811 ± 0.017	0.171 ± 0.055
	×	✓	×	0.748 ± 0.030	1.540 ± 0.346	0.812 ± 0.017	0.174 ± 0.058
	×	×	✓	0.752 ± 0.028	1.536 ± 0.361	0.818 ± 0.018	0.213 ± 0.054
wavesample	✓	×	×	0.749 ± 0.022	1.531 ± 0.325	0.812 ± 0.018	0.170 ± 0.057
	×	✓	×	0.751 ± 0.030	1.542 ± 0.337	0.814 ± 0.017	0.175 ± 0.054
	×	×	✓	0.754 ± 0.028	1.539 ± 0.341	0.819 ± 0.018	0.211 ± 0.055
MSWF	✓	×	×	0.758 ± 0.018	1.386 ± 0.337	0.815 ± 0.019	0.178 ± 0.050
	×	✓	×	0.764 ± 0.020	1.411 ± 0.324	0.819 ± 0.018	0.184 ± 0.055
	×	×	✓	**0.779 ± 0.015**	**1.310 ± 0.313**	**0.824 ± 0.021**	**0.204 ± 0.047**

## Data Availability

These data were derived from the following resources available in the public domain: [OASIS: OASIS-1: Cross-Sectional: https://doi.org/10.1162/jocn.2007.19.9.1498; IXI: https://brain-development.org/ixi-dataset/ (accessed on 1 February 2025)].
